# Higher Responsiveness to Rosuvastatin in Polygenic versus Monogenic Hypercholesterolemia: A Propensity Score Analysis

**DOI:** 10.3390/life10050073

**Published:** 2020-05-20

**Authors:** Agnieszka Mickiewicz, Marta Futema, Agnieszka Ćwiklinska, Agnieszka Kuchta, Maciej Jankowski, Mariusz Kaszubowski, Magdalena Chmara, Bartosz Wasąg, Marcin Fijałkowski, Miłosz Jaguszewski, Steve E. Humphries, Marcin Gruchała

**Affiliations:** 1 Department of Cardiology I, Medical University of Gdansk, Dębinki 7, 80-211 Gdańsk, Poland; marcin.fijalkowski@gumed.edu.pl (M.F.); milosz.jaguszewski@gumed.edu.pl (M.J.); marcin.gruchala@gumed.edu.pl (M.G.); 2 Centre for Heart Muscle Disease, Institute of Cardiovascular Science, University College London, London WC1E 6BT, UK; marta.futema.10@ucl.ac.uk; 3 Department of Clinical Chemistry, Medical University of Gdansk, Dębinki 7, 80-211 Gdańsk, Poland; agnieszka.cwiklinska@gumed.edu.pl (A.Ć.); agnieszka.kuchta@gumed.edu.pl (A.K.); maciej.jankowski@gumed.edu.pl (M.J.); 4 Institute of Statistics, Department of Economic Sciences, Faculty of Management and Economics, Gdansk University of Technology, 80-233 Gdańsk, Poland; mkaszubo@zie.pg.gda.pl; 5 Department of Biology and Genetics, Medical University of Gdansk, Dębinki 1, 80-211 Gdańsk, Poland; mchmara@gumed.edu.pl (M.C.); bwasag@gumed.edu.pl (B.W.); 6 Centre for Cardiovascular Genetics, British Heart Foundation Laboratories, Institute of Cardiovascular Science, the Rayne Building University College London, London WC1E 6JF, UK; steve.humphries@ucl.ac.uk

**Keywords:** polygenic hypercholesterolemia, monogenic hypercholesterolemia, rosuvastatin, propensity score

## Abstract

Background: The monogenic defect in familial hypercholesterolemia (FH) is detected in ∼40% of cases. The majority of mutation-negative patients have a polygenic cause of high LDL-cholesterol (LDL-C). We sought to investigate whether the underlying monogenic or polygenic defect is associated with the response to rosuvastatin. Methods: FH Individuals were tested for mutations in *LDLR* and *APOB* genes. A previously established LDL-C-specific polygenic risk score (PRS) was used to examine the possibility of polygenic hypercholesterolemia in mutation-negative patients. All of the patients received rosuvastatin and they were followed for 8 ± 2 months. A propensity score analysis was performed to evaluate the variables associated with the response to treatment. Results: Monogenic subjects had higher mean (±SD) baseline LDL-C when compared to polygenic (7.6 ± 1.5 mmol/L vs. 6.2 ± 1.2 mmol/L; *p* < 0.001). Adjusted model showed a lower percentage of change in LDL-C after rosuvastatin treatment in monogenic patients vs. polygenic subjects (45.9% vs. 55.4%, *p* < 0.001). The probability of achieving LDL-C targets in monogenic FH was lower than in polygenic subjects (0.075 vs. 0.245, *p* = 0.004). Polygenic patients were more likely to achieve LDL-C goals, as compared to those monogenic (OR 3.28; 95% CI: 1.23–8.72). Conclusion: Our findings indicate an essentially higher responsiveness to rosuvastatin in FH patients with a polygenic cause, as compared to those carrying monogenic mutations.

## 1. Introduction

Familial Hypercholesterolemia (FH) is an inherited lipid disorder affecting roughly one in 250 individuals [1,2]. Long life elevated low density lipoprotein cholesterol (LDL-C) concentrations translate into advanced cardiovascular disease (CVD) [3,4,5]. Clinical criteria are useful for diagnosing FH and selecting patients for genetic testing of three genes coding for proteins that are involved in the clearance of LDL-C from blood: LDL-receptor (*LDLR*), apolipoprotein B (*APOB*), and pro-protein convertase subtilisin kexin 9 (*PCSK9*) [2,6,7,8]. Nevertheless, the mutations in those three genes can only be detected in ∼40% of patients with a clinical diagnosis of FH [4]. It has been previously estimated that a substantial proportion of individuals with clinical phenotype of FH and negative result of FH mutational analysis presents elevated LDL-C concentrations due to a polygenic cause [9]. Those patients inherit a higher than average number of common genetic variants with LDL-C-rising effect and can be identified based on polygenic risk score (PRS) constructed from the top six single nucleotide polymorphisms (SNPs) located in *LDLR, APOB, APOE, ABCG8*, and *SORT1* [10].

Nevertheless, the clinical importance of diagnosing monogenic and polygenic hypercholesterolemia for CV risk assessment and adjusting the intensity of lipid lowering treatment (LLT) remain uncertain [11,12,13,14]. Sharifi et al. showed that the carotid intima media thickness (IMT) and coronary artery calcium (CAC) score, as an indicators of the development of subclinical atherosclerosis, are greater in asymptomatic monogenic FH when comparing to age- and gender- matched asymptomatic polygenic hypercholesterolemia cases [15].

The response to statin therapy has been reported to be related to the genetic basis of FH. In one of the studies, the response to atorvastatin, measured as a mean percentage LDL-C reduction, was significantly higher in heterozygous FH (HeFH) caused by the class 5 mutation in *LDLR* as compared to HeFH individuals with class 2 mutations [16].

Importantly, there is a lack of a comparison of the rosuvastatin efficacy in patients with monogenic vs. polygenic hypercholesterolemia. Thus, the aim of our study was to evaluate the responsiveness to rosuvastatin in patients that were classified as monogenic FH and polygenic hypercholesterolemia. We applied comparative effectiveness analyses while using Inverse Probability Weighted Regression Adjustment (IPWRA) models in order to minimize the possible bias of non-random assignment of the rosuvastatin treatment.

## 2. Materials and Methods

Individuals (n = 112) from outpatient lipid clinic in First Department of Cardiology, Gdansk, Poland, with clinical diagnosis of FH according to validated criteria, were included into the study [17]. The exclusion criteria comprised secondary causes of hypercholesterolemia such as diabetes, hypothyreosis, chronic kidney disease, cholestasis, corticosteroids use as well as the triglyceride (TG) concentration > 4,1 mmol/L.

All of the patients receiving lipid lowering therapy (LLT) were followed for at least six months (mean 8 ± 2) and assessed at least twice during this period. The time points of LDL-C measurements (calculated from Friedewald formula) were defined, as follows: at baseline and before the initiation of rosuvastatin treatment, and after at least six months of LLT.

The patients were enrolled prospectively based on protocol prepared *ad hoc*. The study was reviewed and accepted by the local Ethics Committee (Ethic Codes NKBBN/492/2011-2012 and 362/2019). The informed consent for participation in the study was obtained from all participants. The study complies with the Declaration of Helsinki.

### 2.1. Molecular Testing

Genomic DNA was isolated from whole blood while using standard methods [18] and mutational analysis of *LDLR* and *APOB* was performed in all individuals [19]. A fragment of exon 26 of the *APOB* gene located between codons 3473–3606, which covers the region of the most frequent FH mutation, was screened by using Sanger sequencing [20]. *LDLR* variants were classified into five categories, as indicated by the Association for Clinical Genomic Science, and only individuals with variants that were categorized as class 4 and 5 were diagnosed as monogenic hypercholesterolemia [21].

Mutation-negative patients were genotyped for six LDL-C-raising single nucleotide polymorphisms (SNPs) located in: *CELSR2* (rs629301), *APOB* (rs1367117), *ABCG8* (rs4299376), *LDLR* (rs6511720), and *APOE* (rs429358, and rs7412) at the Cardiovascular Genetics Lab at UCL in the UK with the previously described methods [9,10]. A previously validated LDL-C gene score was calculated for each patient [9,10,22]. Since no control cohort representing the general population is currently available for the PRS analysis, we used quartile cut-offs of the PRS distribution data for the British cohort of Caucasians, the Whitehall II study [9]. The investigated subjects were divided into quartiles of their PRS based on the Whitehall II population. Those with no detectable mutation in *LDLR/APOB* and SNPs in the top 2 quartiles (PRS > 0.65) were defined as polygenic hypercholesterolemia.

### 2.2. Lipid Lowering Therapy

Rosuvastatin was administrated and adjusted in the dose according to the individual decision of physician and patients’ tolerance, aiming to achieve the LDL-C targets based on clinical diagnosis of FH and LDL-C concentrations. The use of ezetimibe was based on the individual decision of the physician. LLT was administrated without any knowledge of the result of genetic testing.

Demographic data, medical history, and parameters associated with LLT, including statin associated muscle symptoms, were evaluated.

High intensity therapy was defined as daily administration of 20–40 mg of rosuvastatin.

We have collected the information about the drug intake by using specially prepared case report form (CRF) to assess the adherence to LLT use.

We assessed previously applied LDL-C goals defined per European Society of Cardiology guidelines [23].

### 2.3. Statistical Analysis

Descriptive statistical evaluations were expressed as numbers and percentages for categorical variables, and as mean with standard deviation (SD) or median with interquartile range (IQR) for continuous variables, when appropriate. The difference between the groups with regards to categorical variables was determined by the Pearson χ^2^ or Fisher exact tests. For continuous variables, the difference between two independent groups was determined by the Welch’s t-test or U Mann–Whitney test, if necessary.

Models have been constructed for two dependent variables to examine the influence of an underlying genetic defect in FH, monogenic or polygenic, on LLT efficacy: percentage of LDL-C reduction (continuous) and LDL-C goal (binomial). The type of hypercholesterolemia (polygenic or monogenic), as well as following variables: age, gender, LDL-baseline, statin intolerance, ezetimibe use, rosuvastatin dose, diabetes, and cardiovascular disease, were examined. The LDL-C goal achievement included reaching both LDL-C targets: in secondary prevention LDL-C < 1.8 mmol/L and in primary prevention LDL-C < 2.5 mmol/L.

The LDL-C reduction variable represented the percentage change of LDL-C after treatment and it was calculated as:

(1) $$LDL-C\;reduction=\frac{LDL-C\;baseline-LDL-C\;after\;treatment}{LDL-C\;baseline}\times 100\%$$

Methods that were conducted for the analysis of LDL-C reduction included: Multiple Linear Regression, ANCOVA (covariance analysis) and Inverse Probability Weighted Regression Adjustment (IPWRA) based on Propensity Score Analysis.

Stepwise analysis with bidirectional elimination method was used to build a multivariate linear regression model. All of the clinically relevant assumptions were tested, namely: normality of residuals (W Shapiro–Wilk test), heteroscedasticity (Breusch-Pagan/Cook-Weisberg test), and multicollinearity (variance inflation factors). Identical set of dependent variables as in regression model was used in covariance analysis (ANCOVA) to calculate the expected marginal means of LDL-C reduction concentration with average values of covariates. An assumption of parallelism was also tested.

The model of discussed relations was based on Potential-outcome models and Propensity Score Analysis (Inverse Probability Weighted Regression Adjustment) to precisely account for non-random assignment to rosuvastatin treatment [24]. A Propensity Score Analysis was implemented while using a multivariable logistic regression model with the use of previous set of variables (the type of hypercholesterolemia—polygenic or monogenic, age, gender, LDL-baseline, statin intolerance, ezetimibe use, rosuvastatin dose, diabetes, and cardiovascular disease) to predict probabilities of conditional treatment assignment on covariates at baseline. Most important for IPWRA, overlap assumption, was verified by density graph of the predicted probabilities (Epanechnikov kernel function). The calculated potential-outcome means (POMeans) as well as frame charts of means with standard errors were presented.

Logistic regression and IPWRA analyses were performed to analyse the achievement of LDL-C goals. The final logistic model was statistically significant as well as each of its independent variables. Additionally, Pearson and Hosmer-Lemeshow goodness of fit test was performed. Outcome model and treatment model were established similarly as the models for LDL-C reduction (%), except the fact that outcome model was logit, not linear. The potential-outcome means (as probabilities of achieving a goal) were calculated.

The level of significance was set at *p* = 0.05. All of the statistical analyses were performed by using Statistica v13.1 (Dell Inc., Round Rock, TX, USA 2016, data analysis software system) and STATA 13 (StataCorp LP, College Station, TX, USA).

## 3. Results

### 3.1. Molecular Testing

Out of the 112 clinical FH subjects, 47 individuals were found to carry an FH-causing variant: 40 (85%) carried a mutation in *LDLR*, and seven (15%) in *APOB* (all *APOB*-mutations were the p.R3527Q). All of the FH-causing variants identified in this study are shown in Supplementary Table S1. The remaining 65 mutation-negative FH patients were genotyped for six LDL-C-associated SNPs. All the mutation-negative patients had a weighted LDL-C PRS above the second quartile of the score distribution, based on the Whitehall II population. These individuals were defined as having a high probability of polygenic cause of hypercholesterolemia. When compared to the Whitehall II population (n = 3020), the PRS in the mutation-negative FH group was significantly higher (0.632 ± 0.22 vs. 0.818 ± 0.09; *p* < 0.001).

### 3.2. Patients’ Clinical Characteristics and Lipid Lowering Therapy Data

The clinical characteristics and comparison of LLT data for monogenic vs. polygenic hypercholesterolemic subjects are shown in Table 1 and Table 2, respectively. The age and gender distributions were comparable in both groups. The individuals positive for *LDLR/APOB* mutations were more often diagnosed with definite FH when compared to the mutation negative patients.

There was no difference in the distribution of cardiovascular risk factors, such as arterial hypertension (HA), smoking, diabetes (DM), and body mass index (BMI) (Table 1). We found a lower frequency of family history of hypercholesterolemia in patients with polygenic hypercholesterolemia than in those carrying the FH-causing mutations. The rate of family history of premature CAD was similar in both groups. Corneal arcus (<45 years of age) was more often present in monogenic hypercholesterolemic subjects than in those with a polygenic cause (*p* = 0.046). Tendinous xanthomas were reported in one patient (Table 1). Carriers of an *LDLR*/*APOB* mutation were found to have higher baseline and post-treatment concentrations of total cholesterol (TC), LDL-C, and non-HDL-C, as compared to patients with polygenic hypercholesterolemia (Table 2). The mean HDL-C and triglyceride (TG) concentrations did not differ significantly between the groups.

The daily doses of rosuvastatin did not differ between the groups (*p* = 0.134). Additionally, 23% of polygenic and 43% of monogenic subjects received ezetimibe (*p* = 0.029). The rates of use of high intensity rosuvastatin therapy and high intensity rosuvastatin in combination with ezetimibe were similar in both of the groups. The distribution of statin intolerance between the groups was comparable (Table 2).

An LDL-C concentration that was below 2.5 mmol/L was achieved in 13% of monogenic and 38% of polygenic subjects (*p* = 0.003). In total, the LDL-C goal was achieved in 31% of polygenic subjects and 11% of monogenic individuals (*p* = 0.012). In primary prevention, individuals with polygenic hypercholesterolemia more frequently reached the goal of therapy (40% vs. 16%; *p* = 0.027). None of the monogenic patients and one patient with polygenic hypercholesterolemia achieved the target of LDL-C below 1.8 mmol/L in secondary prevention (*p* = NS) (Table 2). One polygenic individual in secondary prevention had post-treatment LDL-C of 1.4 mmol/L.

### 3.3. Influence of A Genetic Defect in FH on Response to LLT

**Figure 1:**
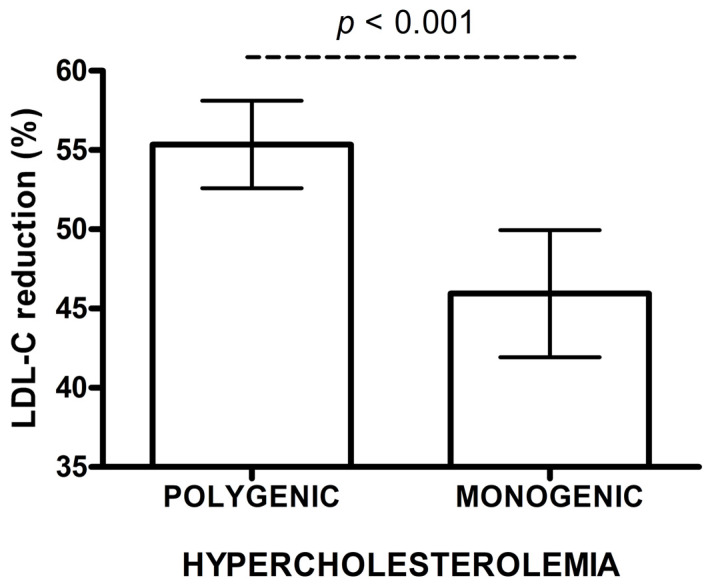
Low density lipoprotein cholesterol (LDL-C) reduction (%) after rosuvastatin treatment in polygenic and monogenic subjects. The data are presented as mean and 95% CI (IPWRA model).

The concentration of baseline LDL-C, and the variability of rosuvastatin doses and ezetimibe use were a major source of bias for the comparison. Thus, we constructed an IPWRA model with a propensity score (PS) to predict the probabilities of treatment assignment conditional on covariates at baseline. After re-weighing of the data, IPWRA model showed a lower percentage of change in LDL-C concentration after treatment in monogenic patients vs. polygenic subjects (45.9% vs. 55.4%, *p* < 0.001) (Figure 1). The ~10 percentage points (pp) difference between the groups in LDL-C change (%) after rosuvastatin therapy was confirmed in covariance analysis (41.5% vs. 51.6%, *p* < 0.001) (Table 3A).

In a linear regression model, the presence of monogenic hypercholesterolemia and the baseline LDL-C concentration substantially influenced the change of LDL-C (%) (*p* < 0.001). Diabetes was a condition with borderline significance (*p* = 0.052). The mean percentage reduction in LDL-C concentration after rosuvastatin in monogenic patients was 10.13 pp lower, as compared to those with polygenic hypercholesterolaemia (*p* < 0.001). Additionally, each increase of baseline LDL-C by 0.02586 mmol/L (1 mg/dL), ceteris paribus, increased the reduction of LDL-C by 0.11 pp. (Table 3A).

### 3.4. Influence of A Genetic Defect in FH on Achieving the LDL-C Treatment Goals

**Figure 2:**
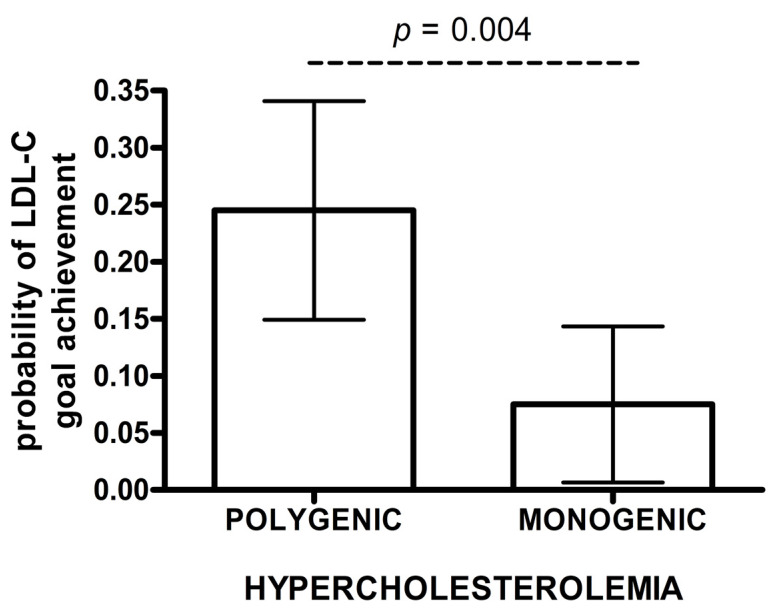
Probability of achieving LDL-C goals after rosuvastatin treatment. The data are presented as mean and 95% CI (IPWRA model).

The estimated probability of achieving LDL-C targets in patients with monogenic hypercholesterolemia was significantly lower than for polygenic subjects in adjusted IPWRA analysis (0.075 vs. 0.245, *p* = 0.004) (Figure 2). Polygenic patients were more likely to achieve the LDL-C treatment goals when compared to monogenic patients (RR = 3.28; 95% CI: 1.23–8.72) (Table 3B). In a logistic regression model, individuals with a polygenic cause of hypercholesterolemia were more likely to achieve LDL-C goals than those with a monogenic cause (OR 3.56, 95% CI: 1.19–10.64). Additionally, a higher proportion of individuals without documented cardiovascular disease achieved LDL-C treatment goals (OR 11.49, 95% CI: 1.46–90.91) (Table 3B).

## 4. Discussion

Intensive LLT is essential in FH individuals to reduce cardiovascular risk and prevent CV death. There is a lack of data on the efficacy of LLT in FH patients with a polygenic cause when compared to individuals carrying a monogenic defect. In our study, we performed a propensity-score weighted analysis of observational data from 112 FH patients in order to evaluate the response to rosuvastatin in those two groups. Unbalanced distribution of LDL-C concentrations, the variability of rosuvastatin doses and ezetimibe use were a major source of bias for the comparison. We found a substantially lower percentage of change in LDL-C concentrations on rosuvastatin therapy in patients carrying the monogenic mutations, in comparison to those mutation-negative with high polygenic score.

Previous studies investigating subjects with polygenic hypercholesterolemia mainly concentrated on the evaluation of CV risk. The treatment response to conventional LLT was mainly assessed in patients with confirmed FH-causing mutations and those with a non-confirmed diagnosis (i.e., no mutation found). To the best of our knowledge, there was no study comparing the response to rosuvastatin in individuals with monogenic FH and polygenic hypercholesterolemia, perhaps due to low availability of polygenic score testing. Data on polygenic hypercholesterolemia are still lacking, as the genotyping for the polygenic score in mutation-negative FH patients is not routinely performed. Therefore, the studies focusing on a treatment response and the achievement of LDL-C goals in polygenic vs. monogenic hypercholesterolemia are valuable in light of new treatment targets and novel drugs [25,26,27,28,29].

The subjects studied here with polygenic hypercholesterolemia and monogenic FH presented a similar frequency of CVD and CV factors, such as hypertension, smoking history, diabetes, and mean BMI. We observed a lower rate of family history of hypercholesterolemia in patients with polygenic hypercholesterolemia than in monogenic patients, which is in line with previous studies. The expected inheritance (i.e., family history) of polygenic hypercholesterolemia is approximately 30%, as compared to the 50% seen in monogenic families [9]. Both groups had a comparable prevalence of clinically probable FH. Our findings indicate that polygenic hypercholesterolemia is sometimes difficult to clinically differentiate from monogenic hypercholesterolemia. The clinical diagnosis of FH cannot clearly identify an underlying genetic defect, which underlines the importance of genetic testing in FH, particularly in probable FH. As NGS becomes cheaper, whole genome sequencing will give individuals a more complete picture of the disease.

In comparison to patients with a high polygenic score, carriers of an *LDLR*/*APOB* mutation were found to have higher mean baseline and post-treatment LDL-C concentrations. The characteristics of our study group is similar to the cohort from Netherlands, in which carriers of FH-causing mutation presented higher baseline LDL-C concentrations and post-treatment LDL-C concentrations, as compared to polygenic subjects [15]. Nevertheless, this study did not provide any detailed information on LLT, the response to treatment, and the achievement of LDL-C treatment goals.

The available data are scarce evaluating the response to rosuvastatin in polygenic and monogenic hypercholesterolemia. One of the studies evaluating the response to evolocumab found no significant difference in percent LDL-C change between those groups after 12 weeks [30]. Although the number of patients was low (32 monogenic vs. 7 polygenic), the authors estimate that it would be needed to analyse 2282 individuals that were treated with evolocumab to observe a significant difference between those groups. Nevertheless, the mechanism of action of evolocumab is different than statins and, thus, we cannot directly compare those findings.

Sijbrands et al. evaluated the response to simvastatin (20 mg per day) in 27 FH patients, for nine weeks and found a similar percentage of LDL-C reduction in patients with confirmed *LDLR* and *APOB* mutations when compared to those with no mutation found [31]. On the other hand, Chaves et al., who investigated the response to statins among patients with different types of mutations in the *LDLR* receptor, found that 22 carriers of *LDLR* null-mutations exhibit a poorer response to simvastatin compared to 20 patients with *LDLR* defective-mutations [32].

Herein, we showed also the low achievement of LDL-C treatment goals. 11% of monogenic subjects when compared to 31% of those with polygenic hypercholesterolemia achieved LDL-C goals, despite a similar distribution of daily doses of rosuvastatin in both groups. Polygenic patients were 3.28 more likely to reach the LDL-C treatment goals as compared to monogenic FH patients. The reason can be explained by higher baseline concentrations of LDL-C in patients with monogenic hypercholesterolemia vs. polygenic subjects along with poorer response to rosuvastatin in patients carrying a monogenic defect.

There are few data on conventional LLT in polygenic and monogenic subjects and we might only compare our results to studies investigating individuals with a confirmed FH-causing mutation compared to those with no mutation found, as we mentioned above [33]. For instance, the Safeheart registry reported that the treatment goal of LDL-C less than 2.5 mmol/L was achieved in only 11.2% of monogenic HeFH [34]. In their study 71.8% of individuals with an FH-causing mutation were on maximal LLT, similarly to our cohort of patients with monogenic hypercholesterolemia (81%). In the large study of Masana et al. only 23% of their patients achieved the target of LDL-C below 2.5 mmol/L, and 12% of them with CVD reached LDL-C concentration of 1.8 mmol/L [27].

Undoubtedly, severe hypercholesterolemia must be treated, irrespective of the underlying genetic cause due to the causal role of elevated LDL-C in the development of atherosclerosis. All of the patients with hypercholesterolemia should reach recommended LDL-C treatment targets to improve their clinical outcome [25,35,36]. However, different approaches can be proposed for monogenic and mutation negative FH cases and in consequence for those with polygenic cause of FH due to higher CV risk in HeFH [37].

The findings of our study highlight a lower rate of the achieved LDL-C targets in patients with monogenic FH as compared to those with polygenic hypercholesterolemia, resulting from higher baseline LDL-C concentrations and poorer responsiveness to LLT and point to the fact that the knowledge about underlying genetic defect in clinically diagnosed FH might be important in terms of patient’s risk stratification and management.

In the era of a personalised medicine approach, when genotype helps to tailor patient’s treatment, and in healthcare systems with limited financial resources, the results of our study would help to identify those patients who will benefit the most from early, intensive therapy with PCSK-9 inhibitors [35,36,38]. Further prospective studies in a larger number of patients with polygenic hypercholesterolemia and monogenic FH would provide more information and evaluate the LDL-C response to oral lipid-lowering medications and PCSK-9 inhibitors, and to assess their CV risk.

### 4.1. Limitations

The limitation of our study is relatively small sample size. However, we would like to underline that polygenic score is not routinely tested yet and the availability of this results is still low. We constructed an IPWRA model based on propensity score and performed covariate analysis to minimize the bias of observational study and evaluate the variables associated with the response to treatment. Nevertheless, further prospective study should be undertaken in patients with clinical phenotype of FH carrying a monogenic defect vs. those with high polygenic score, in order to compare the response to rosuvastatin, as the most potent statin.

Patients were not tested for mutations in *PCSK9*, however the frequency of an FH-causing variant in *PCSK9* in Poland is thought to be even lower than in the UK, where the prevalence is estimated to be 2%. We performed *PCSK9* gene testing in 100 patients with a negative result of *LDLR/APOB* mutational analysis and no *PCSK9* mutations were detected (unpublished data). The whole coding sequence of *APOB* was not analysed. Therefore, we cannot exclude that other mutations, located outside the investigated fragment of exon 26, are present. Nevertheless, the vast majority of pathogenic *APOB* mutations in FH patients are located within exon 26 [21].

### 4.2. Summary

In the absence of randomized data, our comparative effectiveness analysis of observational data with propensity score analysis on the efficacy of LLT can provide guidance for physicians in patients with monogenic FH and polygenic hypercholesterolemia. Within the limitations of an observational study, our findings indicate an essentially higher responsiveness to rosuvastatin in clinical FH patients with a polygenic cause as compared to those with a monogenic cause. The probability of achieving LDL-C targets in patients with monogenic FH was substantially lower than for polygenic hypercholesterolemia subjects. Therefore, we propose closely monitoring FH patients with confirmed monogenic FH-causing mutations in specialized care in order to evaluate their response to LLT and consider an early initiation of PCSK-9 inhibitor therapy.

## Figures and Tables

**Table 1:** Characteristics of individuals with monogenic hypercholesterolemia and polygenic hypercholesterolemia.

Parameter	Polygenic Hypercholesterolemia n = 65	Monogenic Hypercholesterolemia n = 47	*p* Value
Age, years *	54.37 ± 12.54	50.57 ± 13.49	0.134
Female	42 (64.62%)	28 (59.57%)	0.586
Prevention Primary	48 (73.85%)	31 (65.96%)	0.366
Prevention Secondary	17 (26.15%)	16 (34.04%)	0.366
CVD	15 (23.08%)	15 (31.91%)	0.297
CAD	13 (20.00%)	13 (27.66%)	0.343
CAD age, years *	49.45 ± 10.96	47.30 ± 3.56	0.548
MI	21 (32.31%)	8 (17.02%)	0.311
MI age, years *	48.70 ± 11.25	47.38 ± 5.40	0.748
PCI	7 (10.77%)	9 (19.15%)	0.211
PCI age, years *	46.57 ± 12.16	46.78 ± 3.35	0.966
CABG	4 (6.15%)	3 (6.38%)	0.961
CABG age, years *	48.00 ± 5.20	51.5 ± 0.71	0.364
Stroke/TIA	3 (4.62%)	2 (4.26%)	0.748
Smoking	11 (16.92%)	7 (14.89%)	0.783
HA	22 (33.85%)	23 (48.94%)	0.169
DM	6 (9.23%)	2 (4.26%)	0.303
BMI (kg/m^2^)*	26.57 ± 4.11	26.24 ± 4.62	0.720
FH Definite Probable Possible	2 (3.08%) 29 (44.62%) 34 (52.31%)	24 (51.06%) 21 (44.68%) 2 (4.26%)	<0.001 0.995 <0.001
Family history of hypercholesterolemia in adults aged >18 years defined as LDL-C > 4.9 mmol/L (190 mg/dL)	22 (33.85%)	39 (82.98%)	<0.001
Family history of hypercholesterolemia in children defined as LDL-C >4.0 mmol/L (155 mg/dL)	6 (9.23%)	12 (25.53%)	0.021
Family history of premature CAD (in men below age 55, in women below 60 years)	49 (75.38%)	35 (74.47%)	0.912
Corneal arcus <45 y	1 (1.54%)	5 (10.64%)	0.046
Tendinous xanthomata	0	1 (2.13%)	0.420

Data are presented as * mean ± standard deviation or as number (percentage). Abbreviations: BMI—Body mass index, CAD—coronary artery disease, CABG—Coronary artery bypass grafting, CVD—Cardiovascular disease, DM—diabetes, FH—familial hypercholesterolemia, HA—arterial hypertension, MI—myocardial infarction, PCI—percutaneous intervention, TIA—transient ischemic attack.

**Table 2:** Lipid profiles, lipid-lowering therapy and the achievement of LDL-C treatment goals.

Parameter	Polygenic Hypercholesterolemia N = 65	Monogenic Hypercholesterolemia N = 47	*p* Value
Lipid profile parameters before and after treatment			
TC (mmol/L) * baseline after treatment	8.6 ± 1.2 5.0 ± 0.9	10.0 ± 1.8 5.7 ± 1.2	<0.001 <0.001
LDL-C (mmol/L) * baseline after treatment	6.2 ± 1.2 2.9 ± 0.7	7.6 ± 1.5 3.8 ± 1.1	<0.001 <0.001
non HDL-C (mmol/L) * baseline after treatment	7.0 ± 1.4 3.5 ± 0.9	8.3 ± 1.7 4.3 ± 1.1	<0.001 <0.001
TG (mmol/L) * baseline after treatment	1.7 ± 0.8 1.3 ± 0.6	1.6 ± 0.6 1.1 ± 0.5	0.431 0.136
HDL-C (mmol/L) * baseline after treatment	1.6 ± 0.4 1.5 ± 0.4	1.6 ± 0.5 1.5 ± 0.4	0.499 0.421
Lipid lowering therapy			
Rosuvastatin 5–10 mg daily	22 (34%)	9 (19%)	0.134
Rosuvastatin 15–20 mg daily	20 (31%)	22 (47%)	
Rosuvastatin 30–40 mg daily	23 (36%)	16 (34%)	
Ezetimibe use	15 (23%)	20 (43%)	0.029
High intensity rosuvastatin therapy (20–40 mg daily)	41 (63%)	37 (79%)	0.075
High intensity rosuvastatin in combination with ezetimibe	10 (24%)	14 (38%)	0.199
Statin intolerance	7 (11%)	5 (11%)	0.617
SAMS	4 (6%)	4 (9%)	0.451
LDL-C treatment goals			
LDL-C < 2.5 mmol/L	25 (38%)	6 (13%)	0.003
LDL-C < 2.5 mmol/L achieved in primary prevention	19/48 (40%)	5/31 (16%)	0.027
LDL-C < 1.8 mmol/L in secondary prevention	1/17 (6%)	0/16 (0%)	0.515
LDL-C goal achieved	20 (31%)	5 (11%)	0.012

Data are presented as * mean ± standard deviation and number (percentage). Abbreviations: HDL-C-high-density lipoprotein cholesterol, LDL-C—low-density lipoprotein, SAMS—Statin associated muscle symptoms, TC—total cholesterol; TG—triglicerydes.

**Table 3:** Low density lipoprotein cholesterol (LDL-C) reduction and LDL-C goal achievement in monogenic and polygenic hypercholesterolemia.

**A. LDL-C reduction** (**%**)				
**Inverse Probability Weighted Regression Adjustment (IPWRA)**				
**Group**	Mean,% *	*p* value	95% CI	
			lower	upper
**Monogenic**	45.9	<0.001	42.0	49.8
**Polygenic**	55.4		52.7	58.1
**Ancova**				
Group	Mean,% *	*p* value	95% CI	
			lower	upper
**Monogenic**	41.5	<0.001	35.4	47.6
**Polygenic**	51.6		46.6	56.6
**Linear Regression Model**				
**Variable**	F(3,107) = 8.23 *p* < 0.001			
	Coefficient	*p* value	95% CI	
			lower	upper
**Constant**	27.41	<0.001	15.61	39.20
**Monogenic/Polygenic**	−10.13	<0.001	−15.72	−4.54
**LDL baseline**	0.11	<0.001	0.06	0.16
**Diabetes melitus**	−9.38	0.052	−18.84	0.08
**B. LDL-C Goal Achievement**				
**Inverse Probability Weighted Regression Adjustment (IPWRA)**				
**Group**	Probability of goal achievement	*p* value	95% CI	
			lower	upper
**Monogenic**	0.075	0.004	0.008	0.142
**Polygenic**	0.245		0.151	0.339
Risk Ratio Polygenic vs. **Monogenic = 3.28**			1.23	8.72
**Logistic Regression**				
**Variable**	LR Chi^2^ (2) = 16.90 *p* < 0.001			
	Odds ratio	*p* value	95% CI	
			lower	upper
**Constant**	0.625	0.103	0.355	1.100
**Monogenic/Polygenic**	0.281	0.023	0.094	0.840
**CVD**	0.087	0.020	0.011	0.686

Data are presented as * mean and 95% CI or numbers and 95% CI. Abbreviations: see Table 1 and Table 2.

## Supplementary Materials

The following are available online at https://www.mdpi.com/2075-1729/10/5/73/s1, Table S1: Description of *LDLR* and *APOB* mutations in patients with monogenic hypercholesterolemia.
