# Supplementary material for: Higher Responsiveness to Rosuvastatin in Polygenic versus Monogenic Hypercholesterolemia: A Propensity Score Analysis
Source: Life (Basel). 2020 May 20;10(5):73. doi: 10.3390/life10050073 (PMC7281142; doi:10.3390/life10050073)
Supplement: Supplementary file 1 [file life-10-00073-s001.pdf]

SupplementaryMaterials

Higher Responsiveness to Rosuvastatin in Polygenic versus Monogenic Hypercholesterolaemia: A Propensity Score Analysis.

Agnieszka Mickiewicz [1,*], Marta Futema [2], Agnieszka Ćwiklinska [3], Agnieszka Kuchta [3], Maciej Jankowski [3], Mariusz Kaszubowski [4], Magdalena Chmara [5], Bartosz Wasąg [5], Marcin Fijałkowski [1], Miłosz Jaguszewski [1], Steve E. Humphries [6], and Marcin Gruchała [1]

1 Department of Cardiology I, Medical University of Gdansk, Dębinki 7, 80-211 Gdańsk, Poland; marcin.fijalkowski@gumed.edu.pl (M.F.); milosz.jaguszewski@gumed.edu.pl (M.J.); marcin.gruchala@gumed.edu.pl (M.G.)

2 Centre for Heart Muscle Disease, Institute of Cardiovascular Science, University College London, London WC1E 6BT, UK; marta.futema.10@ucl.ac.uk

3 Department of Clinical Chemistry, Medical University of Gdansk, Dębinki 7, 80-211 Gdańsk, Poland; agnieszka.cwiklinska@gumed.edu.pl (A.C.); agnieszka.kuchta@gumed.edu.pl (A.K.); maciej.jankowski@gumed.edu.pl (M.J.)

4 Institute of Statistics, Department of Economic Sciences, Faculty of Management and Economics, Gdansk University of Technology, 80-233 Gdańsk, Polan; agnieszka.kuchta@gumed.edu.pl

5 Department of Biology and Genetics, Medical University of Gdansk, Dębinki 1, 80-211 Gdańsk, Poland; mchmara@gumed.edu.pl (M.C.); bwasag@gumed.edu.pl (B.W.)

6 Centre for Cardiovascular Genetics, British Heart Foundation Laboratories, Institute of Cardiovascular Science, the Rayne Building University College London, London WC1E 6JF, UK; steve.humphries@ucl.ac.uk

* Correspondence: amickiewicz@gumed.edu.pl; Tel.: +48-58-349-25-00; Fax: +48-58-346-12-01

Received: 22 April 2020; Accepted: 16 May 2020; Published: date

Table S1. Description of LDLR and APOB mutations in patients with monogenic hypercholesterolemia.

Gene	*cDNA Change*	*Protein Change*	*Number of Affected Probands*	*ACGS Category*
APOB	c.10580G > A	p.(R3527Q)	7	5
LDLR	c.666C > A	p.(Cys222*)	1	5
LDLR	c.(?_-187)_(190+1_191+1)del	Promoter - exon 2 deletion	2	5
LDLR	c.(67+1_68-1)_(313+1_314-1)del	Exon 2 - exon 3 deletion	1	5
LDLR	c.2056C > T	p.(Gln686*)	1	5
LDLR	c.(313+1_314-1)_(1186+1_1187-1)dup	Exon 4 - exon 8 duplication	4	5
LDLR	c.693C > A	p.(Cys231*)	1	5
LDLR	c.526_533dup	p.(Asp178Glufs*31)	1	5
LDLR	c.1141G > T	p.(Glu381*)	2	5
LDLR	c.1775G > A	p.(Gly592Glu)	11	4
LDLR	c.798T > A	p.(Asp266Glu)	2	4
LDLR	c.1117G > T	p.(Gly373Cys)	1	4
LDLR	c.100T > G	p.(Cys34Gly)	1	4
LDLR	c.2000G > A	p.(Cys667Tyr)	1	4
LDLR	c.662A > G	p.(Asp221Gly)	4	4
LDLR	c.2483delA	p.(Tyr828Phefs*101)	1	4
LDLR	c.265T > C	p.(Cys89Arg)	1	4
LDLR	c.283T > C	p.(Cys95Arg)	1	4
LDLR	c.591C > G	p.(Cys197Trp)	1	4

LDLR	c.530C > T	p.(Ser177Leu)	1	4
LDLR	c.1790C > A	p.(Thr597Asn)	1	4
LDLR	c.191-1G > A	p.?	1	4
